# Spillover effects of military healthcare reform on civilian hospitals in China

**DOI:** 10.3389/fpubh.2025.1702059

**Published:** 2025-11-26

**Authors:** Jie Ma, Yi Zhang, Jianpeng Li, Wenxuan Qu, Zhongliang Zhou

**Affiliations:** 1 Jinhe Center for Economic Research, Xi'an Jiaotong University, Xi'an, China; 2 Department of Cardiovascular Surgery, The First Affiliated Hospital of Xi'an Jiaotong University, Xi'an, China; 3 School of Public Policy and Administration, Xi'an Jiaotong University, Xi'an, China

**Keywords:** military reform, public hospitals, spillover effect, procurement, civilian healthcare

## Abstract

**Background:**

Military hospitals in China served a dual role, providing substantial healthcare to civilians alongside their primary mission of supporting military personnel. The 2015 military reform, which restricted military involvement in civilian services, may have unintentionally reshaped China's healthcare landscape. This study examined the reform's impact on civilian public hospitals, focusing on adjustments in service capacity.

**Methods:**

Using procurement data from China's Government Procurement Database (2015–2021), we applied a difference-in-differences approach to analyze procurement records of civilian public hospitals across cities with differing levels of military healthcare presence. The analysis controls for city-level socioeconomic characteristics. Heterogeneity analyses explore differential effects by geography, procurement size, hospital types, and medical specialty.

**Results:**

Following the reform, civilian hospitals in cities with more military hospitals significantly increased procurement compared to those in cities with fewer military hospitals, particularly after 2019. Specifically, in 2020, the procurement of civilian hospitals in a city with one additional military hospital increased by approximately 43% relative to less-exposed cities, underscoring a significant adjustment. The effect was concentrated in Tier-three hospitals and most pronounced in larger cities and cities located in the Eastern, Western, and Northern Theater Commands. The increase was mainly driven by larger-value purchases (top 50%). Military-specialized procurement (e.g., trauma treatment) showed earlier increases (2018-2019), while non-military specialties (e.g., pediatrics) exhibited delayed responses.

**Conclusion:**

The 2015 military reform inadvertently prompted an expansion of civilian hospital services to absorb displaced patient demand, illustrating the spillover effects of policy interventions in hybrid healthcare systems. These findings underscored the interdependence between military and civilian healthcare infrastructures and demonstrated the utility of procurement data for health policy analysis. Future reforms should consider such cross-system dynamics to minimize unintended spillover effects in access to healthcare.

## 1 Introduction

Military hospitals in China hold a distinctive position at the intersection of national defense and civilian healthcare. Unlike their counterparts in many other countries, which primarily serve active-duty personnel and veterans, Chinese military hospitals have historically provided extensive medical services to civilians as well, particularly following the economic reforms of the 1980s (1). Beyond a few elite institutions ranked among the nation's top care providers, military hospitals comprise a broad network of second- and third-tier facilities across the country. This dual role has made military hospitals indispensable to China's healthcare system (2).

Since 2015, China's military has undergone a broad and systematic reform. A key component was a directive issued by the Central Military Commission in March 2016, requiring the military to withdraw from for-profit civilian activities (3, 4). This sweeping directive was largely motivated by the need to curb widespread abuses, such as illegal sub-contracting and the provision of unapproved treatments in military-run medical facilities (5). The resulting policy imposed a key restriction: limiting military hospitals' expansion in treating civilian patients and redirected their focus toward military medical priorities. While not a complete withdrawal from civilian care, this policy imposed significant constraints, such as caps on the number of hospital beds and limits on recruiting civilian doctors. Given the significant civilian role of these hospitals, such changes were likely to influence civilian healthcare provision and generate spillover effects on civilian hospitals.

Existing literature has documented the integration of military medicine into China's civilian healthcare system. Prior studies examined its specialized training paradigms (6), non-combat operational functions (7), and critical contributions during public health emergencies (8), including the COVID-19 pandemic (9). However, limited research has addressed how large-scale systemic military reforms affect civilian healthcare infrastructure. This represents a significant research gap, particularly given the hybrid nature of China's healthcare system, where public hospitals are state-owned yet operated in a quasi-market environment, competing for both patients and resources.

This study examined the spillover effects of military hospital reform on civilian hospitals in China. To empirically capture the institutional responses of civilian hospitals, we focused on changes in the procurement activity of public hospitals. Procurement records reflect both institutional investments and operational adjustments and are especially informative in the absence of detailed, high-frequency hospital-level performance data (10). Our study focused on public hospitals, which serve the majority of civilian medical needs in China (11). As public institutions, these hospitals are required to follow standardized procurement procedures, and their purchasing activities are recorded in the Chinese Government Procurement Database maintained by the Ministry of Finance. This database contains detailed contract-level information on purchases of medical equipment (e.g., MRI machines, CT scanners, ultrasound systems), medical supplies (e.g., surgical gloves, syringes), and infrastructure services (e.g., facility maintenance and IT systems).

To identify the effects of the military reform, we compared changes in procurement activity among civilian public hospitals across cities with varying numbers of military hospitals, under the assumption that public hospitals in cities with a greater presence of military hospitals were more likely to be affected by the reform. Therefore, differential post-reform trends in procurement across these cities would reflect the reform's spillover impact on civilian hospitals.

Our study made several contributions to the literature. First, it broadened the understanding of the societal impact of military reform by providing new evidence on its unintended spillover effects on civilian public hospitals. Previous literature primarily focused on the internal dimensions of military reform (4, 12, 13). By revealing how the restriction of non-military paid services reshaped the broader healthcare landscape, we showed that reforms aimed at improving military efficiency can have broader, unintended consequences for public service provision.

Second, our study contributed to the literature on China's complex healthcare system. Chinese hospitals represent a hybrid model combining state control with market mechanisms. They are tightly regulated: hospital administrators are often regarded as government officials, required to follow administrative directives, and subjected to price controls on certain medical services. Yet hospitals also compete for patients in a quasi-market environment: patients can freely choose providers, and public health insurance reimburses care regardless of hospital affiliation. Meanwhile, hospitals receive limited operational funding from the government^1^ (14). By leveraging the military reform as a natural experiment, we offered valuable insights into how hospitals behave and interact in this complex healthcare system.

Third, we extended the use of procurement data to the analysis of hospital behavior. Previous research utilizing the Chinese Government Procurement Database primarily focused on government purchasing behavior (15, 16). We demonstrated its potential for capturing institutional responses in the healthcare sector, particularly hospital-level investments and capacity adjustments. This complemented earlier work such as Rahal (17), who highlighted the value of linking procurement payment data to healthcare institutions in the UK. To our knowledge, this study is among the first to apply such data to analyze hospital behavior. Furthermore, procurement data provide a novel lens compared to commonly used public health insurance records. Whereas insurance data capture diagnoses, treatments, and expenditures at the patient level, procurement data directly reflect hospitals' strategic decisions related to infrastructure and service provision. This approach enhances our understanding of hospital behavior, especially in contexts where detailed clinical or administrative data are scarce, and holds potential for broader application in other countries with substantial public healthcare systems.

In this study, we pursued two objectives. First, we assessed whether civilian hospitals responded to the redirection of medical demand by expanding their institutional capacity. Such adjustments could involve hiring additional staff, increasing the number of available hospital beds, or expanding procurement activity. In this study, we focused on changes in their procurement activity. Second, we explored the heterogeneity in this response across multiple dimensions. Military medicine traditionally emphasized areas distinct from civilian healthcare, such as trauma surgery, mental health care (e.g. PTSD management), preventive care in extreme environments, and disaster response (6). We tested whether civilian hospitals experienced more pronounced growth in these military-oriented specializations following the reform. We also analyzed heterogeneity across hospital characteristics, procurement types, and city-level factors to gain more systematic insights.

## 2 Materials and methods

### 2.1 Data

Our primary data source is the public procurement payment records from the China Government Procurement Website.^2^ The database provides comprehensive coverage of procurement contracts issued by all levels of government and public institutions. These publicly available records include detailed information on contract titles, procured items, types of goods or services, purchasing agencies, contractors, transaction amounts, contract dates, and winning bidders. We used data from 2015 to 2021. The year 2015 marks the beginning of consistent nationwide disclosure of procurement data, while extending the sample to 2021 allows us to capture the full dynamic adjustment of public hospitals in the years following the announcement and implementation of the military reform.

#### 2.1.1 Outcome variable: civilian hospital procurement

Following existing studies using this dataset, we employed web crawlers to extract procurement records related to public hospitals. To identify relevant records, we first compiled a comprehensive list of civilian public hospitals from the National Health Insurance Service Platform for Designated Medical Institutions.^3^ This database lists all hospitals recognized by China's public health insurance system and provides information on their locations, city tiers, and ownership types (public or private). We focused on public hospitals, which are expected to appear in the procurement database. As of 2013, public hospitals accounted for 84.4 percent of total hospital beds, employed 85.8 percent of all hospital health professionals, provided nearly 90 percent of inpatient and outpatient services, and were responsible for about 70 percent of total hospital expenditures (18). Thus, public hospitals reflect the majority of civilian healthcare adjustments. We further restricted our sample to second- and third-tier hospitals, which represent the core of China's hospital infrastructure and are more comparable to military hospitals.

To link the hospital list with the procurement records, we applied fuzzy matching between hospital names in our list and the names of purchasing institutions in the procurement data using the “fuzzywuzzy” Python package. Matches with a similarity score of 80% or higher were kept and then manually verified for accuracy. To mitigate the influence of outliers, we winsorized purchase amounts at the 99th percentile of the distribution. The resulting dataset comprised 59,174 valid hospital procurement records from 2015 to 2021.

Our main outcome variable, *y*_*it*_ is the logarithm of the total procurement amount by civilian public hospitals in city *i* and year *t*. We constructed it by aggregating the individual procurement records to the city-year level, yielding 877 observations across 228 cities over the sample period. If a city had no procurement records in a given year and all prior years, we treated the observation as missing, reflecting possible gaps in early data coverage due to the gradual adoption of public procurement practices. However, if prior records existed but none appeared in a later year, we coded the later observation as zero. We chose the logarithmic transformation primarily because the procurement variable is right-skewed, so the transformation helps to normalize the distribution of the variable, improving the accuracy and reliability of the regression estimates. Furthermore, using the log of procurement allows the estimated coefficients to be directly interpreted as the percentage increase in civilian procurement following the reform, offering a more intuitive and economically meaningful measure of the effect.

#### 2.1.2 Treatment variable: military hospital presence

To capture regional variation in exposure to the reform, we used the number of military hospitals in each city, *MH*_*i*_. The underlying logic is that cities with a greater military hospital presence were more affected by the reform, as the withdrawal of these facilities from civilian services created a larger local demand shock. Using the aforementioned hospital list from the National Health Insurance Service Platform, we identified military hospitals by searching for keywords indicating military affiliation (e.g., “Jun” for military or “Budui” for troops) in their names, followed by manual verification to ensure accuracy. The variable *MH*_*i*_ was constructed as a time-invariant city-level count, reflecting the number of military hospitals present during the study period. We did not track changes over time, as hospital establishments tend to be stable in the short term and annual changes were minimal and inconsistently recorded.

#### 2.1.3 Control variables

We controlled for a range of city-level characteristics that could affect hospital procurement and local healthcare demand. These controls include indicators of local economic development, such as GDP per capita, fiscal expenditure, and city tier ranking, as well as demographic characteristics, including population size and age structure (percentage age >65, percentage age < 14). Among these variables, GDP per capita, fiscal expenditure, and population size were log-transformed to address right-skewed distributions. Data for these control variables were mainly drawn from the China City Statistical Yearbook. When data were missing from the yearbooks, we supplemented with the China Stock Market & Accounting Research Database (CSMAR) and apply linear interpolation where necessary. City tier rankings were obtained from the annual “City Business Attractiveness Ranking” published by Yicai Magazine,^4^ a widely used classification in the literature (19).

### 2.2 Empirical model

To examine the impact of the military reform on civilian public hospitals, our empirical approach compares the post-reform trend in procurement in highly-exposed cities (those with high *MH*_*i*_) to the trend in less-exposed cities (those with low or zero *MH*_*i*_). All econometric analyses were performed using Stata.The econometric model is presented below:


$$\begin{array}{l} y_{it}=\beta_{1}+\beta_{2}MH_{i}+\underset{t}{\sum }\beta_{t}\left({\text{year}}_{t}\cdot MH_{i}\right)+{\text{year}}_{t}+X_{it}\gamma \\ \;+province_{i}+\epsilon_{it}. \end{array}$$

The main outcome variable *y*_*it*_ is the logarithm of the total procurement amount by civilian public hospitals in city *i* and year *t*. The variable *MH*_*i*_ represents the number of military hospitals in city *i*.

Our key explanatory variables are the interaction terms between year dummies and *MH*_*i*_. The coefficients β_*t*_ capture the differential changes in procurement activity over time across cities with different levels of military hospital presence. Since the dependent variable is in logarithmic form, β_*t*_ represents the percentage change in procurement amount associated with one additional military hospital in a city. A significant, positive β_*t*_ in these post-reform years would indicate that cities with greater military hospital exposure experienced larger increases in public hospital procurement relative to less-exposed cities.

The vector of control variables, *X*_*it*_, include log-transformed city-level socioeconomic indicators (population, GDP per capita, fiscal expenditure), city tier fixed effects, and population age structure (percentage age>65, percentage age < 14). We included year fixed effects (*year*_*t*_) to control for national-level policy changes or macroeconomic shocks affecting all cities, and province fixed effects (*province*_*i*_) to account for time-invariant regional characteristics across provinces, such as baseline healthcare infrastructure or governance quality^5^. Robust standard errors were clustered at the city level to account for serial correlation and heteroskedasticity within cities over time.

#### 2.2.1 Heterogeneity analyses methodology

To provide a comprehensive assessment, we conducted a series of heterogeneity analyses focusing on three dimensions: geographic factors, procurement characteristics, and hospital type. All analyses were performed by re-estimating the baseline model on relevant sub-samples or using disaggregated procurement variables.

Since 2016, the military forces in China have been divided into five theater commands: Eastern (Jiangsu, Zhejiang, Anhui, Fujian, Jiangxi, Shanghai), Southern (Guangdong, Guangxi, Hainan, Hunan, Yunnan, Guizhou), Western (Sichuan, Chongqing, Shaanxi, Gansu, Ningxia, Qinghai, Xinjiang, Tibet), Northern (Liaoning, Jilin, Heilongjiang, Shandong, Inner Mongolia), and Central (Beijing, Tianjin, Hebei, Shanxi, Henan, Hubei). For the regional analysis, we classified cities according to China's post-2016 theater command structure (Eastern, Southern, Western, Northern, and Central) to assess whether variations in military governance and reform implementation intensity across commands influenced the magnitude of market shocks. For city scale, we grouped cities as above Tier 3 (larger) and at or below Tier 4 (smaller) to test whether the hospital response was concentrated in large, crowded urban markets where military hospitals were typically located and demand absorption opportunities were highest.

For procurement characteristics, we analyzed both procurement size (classified into quartiles, e.g., Top 10% to Bottom 50%) and procurement content type. The size analysis tested if the reform induced systemic restructuring reflected in high-value capital expenditures versus low-value routine purchases. The type analysis distinguished between medical supplies (routine costs) and medical devices (capital investments), as well as between military-related and non-military-related specialties. This specialty breakdown allowed us to identify whether civilian hospitals initially focused their expansion on absorbing displaced demand in military-focused services before diversifying into broader medical fields. Finally, to explore heterogeneity by hospital tier, we separated procurement records for Tier-Two and Tier-Three hospitals to assess whether the reform effect was predominantly concentrated in the largest, most advanced Tier-Three hospitals, which were more likely to absorb demand from divested military facilities.

## 3 Results

Our study is a quasi-experimental observational study using secondary data. We compared changes in procurement across cities with different levels of military hospital presence, treating cities with fewer or no military hospitals as the control group. We first presented the descriptive statistics for cities with and without military hospitals. Then we presented the results addressing our two main research objectives: the impact of military hospital presence on civilian hospital procurement and the heterogeneity across regions, hospital tiers, and procurement characteristics.

### 3.1 Descriptive statistics

Table 1 presents summary statistics for variables used in the analysis. The left panel shows data for cities without military hospitals (military = 0), which is our control group minimally affected by the direct supply shock. The right panel show data for those cities with at least one military hospital (military>0), the treatment group exposed to redirected medical demand following the reform. On average, cities with military hospitals had significantly higher levels of civilian public hospital procurement during the sample period. The large standard deviations in procurement values are likely attributed to the wide heterogeneity in city scale, the lumpy nature of hospital capital expenditures, and the progressive disclosure of public procurement records. The inclusion of city and year fixed effects helps account for these baseline variations and volatility.

**Table 1:** Summary statistics of variables by military presence.

	**Military** = **0**					**Military >0**				
**Variable**	**Obs**	**Mean**	**Std. Dev**.	**Min**	**Max**	**Obs**	**Mean**	**Std. Dev**.	**Min**	**Max**
Procurement	533	4,820.193	8,492.138	0.000	82,334.220	344	17,855.790	42,580.130	0.000	383,426.600
Population	443	290.719	211.957	20.379	1,077.280	344	621.626	533.032	40.169	3,416.290
GDP per capita	443	51,900.660	36,845.510	10,527.000	279,499.400	344	76,119.960	38,734.600	20,577.000	187,415.000
Fiscal budget	443	375.022	543.733	24.274	4,593.800	344	1,088.705	1,511.936	110.195	8,351.540
Percentage of pop age>65	533	0.111	0.024	0.071	0.188	344	0.125	0.028	0.072	0.188
Percentage of pop age < 14	533	0.188	0.024	0.108	0.236	344	0.170	0.038	0.093	0.241
Tier-three hospitals	533	5.649	7.618	0.000	57.000	344	27.395	28.357	1.000	134.000
Tier-two hospitals	533	29.841	22.428	1.000	117.000	344	73.587	65.254	5.000	424.000
Number of public hospital beds	368	13,910.330	9,798.505	1,465.000	63,276.000	323	39,400.350	34,602.860	2,361.000	181,005.000
Number of public doctors	368	7,402.573	6,513.831	844.000	44,309.000	323	22,503.130	20,763.200	2,162.000	122,166.000
Number of public hospitals	533	146.310	121.073	5.000	592.000	344	477.663	1,272.476	34.000	9,161.000

Treatment cities also tended to be larger, with higher population, fiscal expenditure, and slightly older populations. While GDP per capita was somewhat higher in cities with military hospitals, the difference was relatively modest, suggesting that these cities were generally more populous and better resourced, but not necessarily much wealthier per capita. In terms of healthcare infrastructure, cities with military hospitals had more hospitals overall, particularly more second- and third-tier hospitals, and significantly larger numbers of hospital beds and doctors. These differences highlighted the systematic variation in city size and healthcare capacity associated with the presence of military hospitals, providing important context for interpreting the impact of the reform.

Appendix Figure 1 in the Appendix illustrates the geographic distribution of military hospitals across Chinese cities, showing substantial regional variation. Most provinces hosted at least one military hospital, with southeastern coastal provinces having a higher concentration than western and remote regions. Within provinces, military hospitals were typically located in economically developed and densely populated cities. An exception to this pattern was Xinjiang, a western province that hosted a relatively high number of military hospitals. This was likely due to its strategic geopolitical significance and critical role in national security. Moreover, the distribution of military hospitals also aligned with the historical layout of China's military command structure, with major hospitals located in cities that had served as headquarters for key military regions such as Beijing, Jiangsu, Sichuan, and Guangdong. Overall, these patterns suggest that both strategic military priorities and urban development levels significantly shaped the geographic placement of military hospitals.

### 3.2 Impact of military hospital presence on civilian hospital procurement

Table 2 presents the main estimates of the reform's impact on civilian public hospital procurement. The dependent variable is the logarithm of total civilian procurement, allowing the coefficients to be interpreted as the percentage change. Columns (1) through (6) show results from different specifications, progressively adding controls. The presence of the “X” symbol in the table indicates the inclusion of control variables and/or fixed effects. Coefficients for these control variables are omitted from the table to maintain focus on the main coefficients of interest and avoid excessive length.

**Table 2:** Main results.

	**Log procurement**				
	**(1)**	**(2)**	**(3)**	**(4)**	**(5)**
	**Baseline**	**FE**	**Basic controls**	**Full spec**	**More controls**
Military Hospital	0.204^***^	0.306^*^	−0.136	−0.135	−0.120
	(0.0618)	(0.160)	(0.204)	(0.201)	(0.205)
2016^*^Military Hospital	0.129	0.149	0.133	0.145	0.143
	(0.102)	(0.114)	(0.110)	(0.105)	(0.105)
2017^*^Military Hospital	0.306^**^	0.245^*^	0.201	0.217^*^	0.211^*^
	(0.124)	(0.133)	(0.124)	(0.124)	(0.121)
2018^*^Military Hospital	0.287^**^	0.0819	0.0683	0.0548	0.0449
	(0.111)	(0.111)	(0.111)	(0.113)	(0.115)
2019^*^Military Hospital	0.252^**^	0.220^*^	0.229^*^	0.225	0.238^*^
	(0.124)	(0.132)	(0.134)	(0.141)	(0.143)
2020^*^Military Hospital	0.282^**^	0.435^***^	0.463^***^	0.447^**^	0.459^**^
	(0.122)	(0.160)	(0.172)	(0.174)	(0.180)
2021^*^Military Hospital	0.103	0.332^**^	0.338^**^	0.344^**^	0.367^**^
	(0.146)	(0.154)	(0.155)	(0.158)	(0.163)
City controls			X	X	X
Population Age ratio				X	X
Year FE		X	X	X	X
Province FE		X	X	X	X
Has Regional Center					X
N	877	877	691	691	691
adj. R-sq	0.046	0.439	0.445	0.447	0.448

The outcome variable is the logarithm of the aggregated yearly procurement amount by civilian public hospitals in each city. Military Hospital represents the number of military hospitals in a city. Standard errors clustered at the city level are reported in parentheses. ^***^p < 0.01, ^**^p < 0.05, ^*^p < 0.10.

Column (1) reports the baseline relationship between the number of military hospitals and civilian hospital procurement. The positive coefficient aligns with the descriptive evidence in Table 1, indicating that cities with more military hospitals tended to have higher procurement volumes. The key coefficients are the interaction terms between year fixed effects and the number of military hospitals. Without additional controls, these interactions are positive and statistically significant in later years, suggesting that cities with more military hospitals experienced larger increases in procurement. In Column (2), we introduced year fixed effects and province fixed effects, which absorbed common time trends and time-invariant differences across provinces. As a result, the R-squared increases from 0.046 to 0.439, suggesting a large improvement in the model fit. The coefficients of the interaction terms remain positive and statistically significant in the later years, particularly in 2020 and 2021. In 2020, cities with one more military hospital experienced a 43.5% increase in procurement.

Columns (3) to (4) added city-level control variables to account for potential confounding factors. Column (3) controlled for population, GDP per capita, fiscal expenditure, and city tier dummies. Column (4) further included the percentage of the population aged over 65 and under 14. The interactions in the later years remain positive and significant across all specifications, underscoring the robustness of the results. In later analyses, we used the model in Column (4) as our main specification, unless otherwise specified.

We also considered other medical reforms that occurred during the same period. One such policy was the introduction of national-level regional medical centers, designated to act as regional hubs that strengthened healthcare service capacity and improved resource allocation. Five waves of such centers were implemented between 2019 and 2023. Column (5) accounted for whether a city hosted a regional medical center. By including this variable, we accounted for potential procurement increases driven by this policy rather than the military hospital presence. The results remain consistent.

These findings suggest that civilian hospital procurement had experienced significantly larger increases in cities with a greater presence of military hospitals, especially in later years following the reform. As military hospitals reduced civilian services, public hospitals expanded to absorb the resulting unmet demand. The effects intensified over time and peaked in 2020 and 2021, reflecting a dynamic adjustment process. This delayed response likely resulted from the accumulated restriction of military hospitals over time and structural rigidity in expanding healthcare capacity, as investments in infrastructure and personnel required time. It should also be noted that the observed increase in 2020 and 2021 coincided with the COVID-19 pandemic, which may have further intensified demand and accelerated the expansion of civilian hospital services.

The City Statistical Yearbook also provides data on healthcare infrastructure, including the number of hospitals, second- and third-tier hospitals, hospital beds, and doctors. These indicators include all public hospitals and could have potentially experienced expansions resulting from the military reform. Therefore, we did not control for public medical resources in the main regression. Instead, we used them as additional outcome variables to study overall changes in public medical resources.

To test the robustness of our results, we re-estimated the model using data on the number of public hospitals, hospital beds, and doctors. The results were reported in Appendix Table 1. We also separated the number of tier-three and tier-two hospitals as outcomes. However, the coefficients on the interactions between year dummies and military hospitals were not statistically significant. One possible explanation is that the number of hospitals reflects the stock of the public medical system, while procurement captures the year-to-year changes. As a result, procurement data are better suited to detect the impact of policy shifts or reforms. This difference is reflected in the R-squared values: the regressions using healthcare resource stocks (Appendix Table 1) yielded R-squared values around 0.95, indicating limited variation after accounting for fixed effects, whereas those for procurement data (Table 2) were about 0.4, reflecting greater responsiveness over time. Additionally, there were notable missing data in the city-level statistic yearbooks regarding public healthcare resources: some cities did not update these data annually, requiring us to apply linear interpolation for these cases. This limitation may have further limited their reliability as indicators of short-term change.

### 3.3 Heterogeneous impacts of the reform

This subsection explores the heterogeneous impacts of the reform across market, geographic, and institutional dimensions, consistent with the methodology outlined in Section 2.

#### 3.3.1 Heterogeneity by region and city scale

Table 3 reported the heterogeneity in treatment effects across different regions and city tiers. For the regional analysis, we classified cities according to China's post-2016 theater command structure. The results showed that the reform effects were primarily concentrated in cities in the Eastern, Western, and Northern Theater Commands, while cities in the Southern and Central Commands exhibited weaker or insignificant effects. This pattern likely reflected variation in the intensity and pace of reform implementation across military regions, each of which operated under a separate command structure. The decentralized nature of military governance may have led to differences in execution strategy, prioritization, or enforcement strength across commands.^6^

**Table 3:** Heterogeneity by city types.

	**(1)**	**(2)**	**(3)**	**(4)**	**(5)**	**(6)**	**(7)**
	**East**	**Center**	**North**	**South**	**West**	**Large cities**	**Small cities**
2016^*^Military hospital	0.858^***^	0.176^***^	−0.140	−0.568	0.126	−0.106	0.480
	(0.316)	(0.0326)	(1.035)	(0.447)	(0.176)	(0.366)	(1.011)
2017^*^Military hospital	1.444^***^	0.267^***^	2.919	0.391	0.240	0.289	0.00682
	(0.307)	(0.0866)	(1.960)	(0.571)	(0.251)	(0.189)	(1.143)
2018^*^Military hospital	1.335^***^	0.269^***^	5.329^**^	−0.614	−0.107	0.138	−0.135
	(0.410)	(0.0829)	(2.333)	(0.516)	(0.119)	(0.190)	(1.024)
2019^*^Military hospital	1.841^***^	0.241^***^	5.600^**^	−0.343	−0.157	0.736^***^	−0.909
	(0.651)	(0.0841)	(2.458)	(0.725)	(0.213)	(0.277)	(1.092)
2020^*^Military hospital	2.005^***^	0.157^*^	7.246^***^	0.676	−0.237	0.977^***^	−0.602
	(0.584)	(0.0840)	(2.285)	(0.606)	(0.256)	(0.323)	(1.105)
2021^*^Military hospital	1.555^**^	0.184	6.569^***^	0.159	−0.324	0.447	0.129
	(0.730)	(0.138)	(2.224)	(0.711)	(0.243)	(0.326)	(1.201)
N	188	89	94	176	144	313	353
adj. R-sq	0.469	0.522	0.513	0.459	0.574	0.403	0.536

The outcome variable is the logarithm of the aggregated yearly procurement amount by civilian public hospitals in each city. Military Hospital represented the number of military hospitals in a city. Columns (1)–(5) presented results for China's five theater commands: ETC (Eastern Theater Command), WTC (Western Theater Command), CTC (Central Theater Command), NTC (Northern Theater Command), and STC (Southern Theater Command). Columns (6) and (7) presented results for different city tiers: Column (6) included cities in Tier 1.5, Tier 2 and Tier 3 which are largest cities in China. Column (7) included the rest of the smaller cities. All specifications include province fixed effects and city level control variables. Standard errors clustered at the city level are reported in parentheses. ^***^p < 0.01, ^**^p < 0.05, ^*^p < 0.10.

With respect to city tiers, we divided the cities into two groups. One included cities above Tier 3, which were the larger cities in China. We excluded the five largest cities in Tier 1: they all had military hospitals, but no cities without military hospitals were comparable to them in terms of size, population, or economic development. The other group included cities at or below Tier 4, which represented the smaller cities in China. The estimated effects of the reform were concentrated in larger cities and were insignificant in smaller cities. These results could be driven by the fact that military hospitals were concentrated in larger cities, as shown in Table 1. Hospitals in top-tier cities were already operating under crowded conditions before the reform (20). Therefore, after the reform, public hospitals in these cities had an opportunity to expand and responded more actively to absorb the displaced demand.

#### 3.3.2 Heterogeneity by procurement characteristics

Next, we explored heterogeneity in the reform effects by different types of procurement, focusing specifically on procurement size. Our goal was to assess whether the impact of the reform was more pronounced for larger or smaller procurement transactions. To do this, we returned to the original procurement records and ranked all purchases within each year. We then classified these transactions into four groups based on their rank in each city-year: the top 10%, 10–25%, 25–50%, and bottom 50%. For each group, we calculated the total procurement amount and estimated the regressions separately.

Appendix Table 2 in the appendix reported the results. The effects of the reform were less statistically significant in the bottom 50% procurement group, suggesting that smaller procurement transactions were less affected. However, the magnitudes of the coefficients were mostly comparable to those in other groups. In contrast, the effects were more consistently significant for higher-value procurements, where larger transactions may better capture systemic shifts in hospital purchasing behavior. This pattern was consistent with the idea that the reform prompted more substantial restructuring and expansion efforts in response to major procurement needs, while routine or low-value purchases remained less sensitive to institutional changes.

We further investigated the heterogeneity of the reform's effects by examining procurement types in terms of product content and medical specialty. First, we categorized procurement by product content into two groups: medical supplies, typically lower-cost items requiring frequent repurchasing, and medical devices, which were more expensive and procured less frequently. Second, we classified procurement by medical specialty into two categories: military-related specialties (e.g., trauma surgery, mental health, emergency care), which were traditionally prioritized in military medicine (21), and non-military-related specialties (e.g., obstetrics, gynecology, pediatrics), which fell outside the core scope of military medicine. To implement these categorizations, we relied on the official catalog of medical materials published by the State Drug Administration,^7^ which classified medical products by type and specialty. We then manually matched each procurement record in our dataset to the corresponding product and specialty categories.

The results were presented in Table 4. Columns (1) and (2) reported the effects of the reform on the procurement of medical supplies and devices, respectively. While neither category showed consistently significant effects, the coefficients for medical supplies were more consistently positive and larger in magnitude. This suggested that hospitals might have been somewhat more responsive in adjusting routine supply purchases, which were generally more flexible than equipment investments. Columns (3) and (4) showed the results for military and non-military specialties. We hypothesized that the reform would have a stronger impact on military specialties, as they were more directly affected by the restructuring of military hospitals. The results provided partial support: although the overall differences between the two groups were not substantial in terms of coefficient size or significance, we observed some temporal variation. Procurement in military specialties showed an earlier response in 2018–2019, which diminished in later years, whereas non-military specialties exhibited more delayed effects, primarily appearing in 2019–2020. This staggered adjustment may have reflected a two-phase response: initially, civilian hospitals absorbed demand in military-related areas directly impacted by the reform, followed by a broader expansion in civilian specialties as displaced patients gradually shifted across service categories.

**Table 4:** Heterogeneity by procurement category and hospital tiers.

	**(1)**	**(2)**	**(3)**	**(4)**	**(5)**	**(6)**
	**Material**	**Equipment**	**Advantage**	**Disadvantage**	**Tier-two**	**Tier-three**
2016^*^Military hospital	−0.0599	−0.0823^**^	−0.0600	0.0546	−0.0538	0.106
	(0.181)	(0.0344)	(0.0656)	(0.0590)	(0.0802)	(0.0859)
2017^*^Military hospital	−0.145	−0.0429	0.0331	0.0370	0.0701	0.171^*^
	(0.0947)	(0.0651)	(0.0490)	(0.0701)	(0.0547)	(0.0990)
2018^*^Military hospital	0.0818	−0.00783	0.161^***^	0.105	0.00164	0.187^*^
	(0.104)	(0.0597)	(0.0551)	(0.102)	(0.0533)	(0.0956)
2019^*^Military hospital	0.138	−0.0196	0.107^*^	0.0789	0.0307	0.172^*^
	(0.124)	(0.0675)	(0.0630)	(0.0762)	(0.0688)	(0.0978)
2020^*^Military hospital	0.0734	0.0332	0.0632	0.132^**^	0.0502	0.168^*^
	(0.135)	(0.0595)	(0.0550)	(0.0512)	(0.0551)	(0.0898)
2021^*^Military hospital	0	0.0173	0.00935	0.110	−0.117^*^	0.0707
	(.)	(0.0614)	(0.0842)	(0.0709)	(0.0696)	(0.0905)
N	164	321	280	290	433	444
adj. R-sq	0.148	0.481	0.457	0.476	0.484	0.414

The outcome variable is the logarithm of the aggregated yearly procurement amount by civilian public hospitals in each city. Military Hospital represents the number of military hospitals in a city. Columns (1) and (2) presented results for procurement of medical supplies and devices, respectively. Columns (3) and (4) presented results for procurement of supplies related to military medical specialties (e.g., trauma surgery, mental health, emergency care) and non-military medical specialties (e.g., obstetrics, gynecology, pediatrics). Columns (5) and (6) presented results for procurement of Tier-two hospitals and Tier-three hospitals. All specifications include province fixed effects and city-level control variables. Standard errors clustered at the city level are reported in parentheses. ^***^p < 0.01, ^**^p < 0.05, ^*^p < 0.10.

#### 3.3.3 Heterogeneity by hospital type

We also explored whether the effects differed by hospital type. We separated procurement by tier-two and tier-three hospitals and calculated the total procurement amount for each type at the city-year level. The results were reported in Columns (5) and (6) of Table 4. The coefficients for third-tier hospitals were positive and statistically significant, while those for second-tier hospitals were smaller in magnitude and statistically insignificant-with the only significant coefficient being negative. These findings suggested that the effect of the military reform was concentrated in the third-tier hospitals, which were the largest and most advanced hospitals in China. This may have been because military hospitals were themselves typically large and high-standard facilities, making them more comparable to third-tier hospitals. Therefore, displaced demand from military hospitals was likely absorbed primarily by third-tier hospitals.

## 4 Discussion

This study examined the effect of China's 2015 military reform on the development of civilian public hospitals. Specifically, we pursued two objectives: first, to assess whether civilian hospitals responded to the redirection of medical demand by expanding their institutional capacity, with a focus on changes in procurement activity; and second, to explore the heterogeneity of this response across medical specialties (particularly military-oriented ones), hospital characteristics, procurement types, and city-level factors.

Using procurement records from 2015 to 2021, we found that procurement by civilian hospitals increased significantly in cities with a greater presence of military hospitals, particularly in the later years. These results suggested that civilian hospitals expanded to accommodate the displaced demand previously served by military hospitals. Heterogeneity analyses revealed meaningful variation by region, procurement scale, and medical specialty. We found that the effect was concentrated in Tier-three hospitals, which were most comparable to military hospitals, typically high-quality general hospitals. The effect was concentrated in larger, mid-tier cities (above Tier 3) and the growth was driven by higher-value procurement transactions (top 50%). Procurement for military-related specialties also showed an earlier response (2018–2019) than non-military specialties.

Existing literature has documented the role of military medicine in peacetime settings. While its core mission has been to treat combat-related injuries, the influence of military medicine extends far beyond combat, with the majority of clinical activities originating from garrison-based care and services for veterans (22). Moreover, civilian healthcare systems have long benefited from military-led medical advances, particularly in trauma surgery, pandemic response, and mental health such as post-traumatic stress disorder (PTSD) research (21, 23). Military medical readiness is also maintained through continuous training and collaboration with civilian trauma centers (21, 24). Our study provides quantitative evidence on the dynamic interdependency of these two sectors, showing that civilian hospitals, particularly high-tier facilities, actively absorbed displaced demand.

### 4.1 Policy implication

Our findings offered important policy implications for healthcare governance and reform. First, reforms targeting military efficiency can generate substantial spillover effects in the broader healthcare system. Cheng (25) examined the impact of military reforms before 2005 which was institutionally distinct, and a consistent theme emerged: military reform can reshape both military and civilian healthcare. Future reforms should adopt a system-wide perspective, anticipating cross-sector impacts and ensuring smooth transitions. This is particularly relevant in other countries where military healthcare also serves civilians (24).

Second, the reform's effects varied across Theater Commands, indicating regional disparities in reform implementation. The heterogeneity likely stemmed from differences in command-level priorities, enforcement, and the extent of local reliance on military medical services. Pei et al. (26) highlighted challenges in adapting to the newly established theater commands during the military reform. Policymakers should aim for standardizing reform objectives across regions while providing targeted support to areas historically more reliant on military hospitals.

Third, procurement growth was most significant in larger cities, where military hospitals were more prevalent. Although we excluded the largest 4 megacities (Beijing, Shanghai, Shenzhen, and Guangzhou) from the city tier analyses, the effects of the reform for these megacities were in fact negative. This suggested a possibility that the military reform may have helped move some of the healthcare capacity from the largest cities to the slightly smaller cities. These cities likely had more flexibility to expand and serve redirected demand. Great efforts have been made to better reform China's public hospitals to improve healthcare accessibility and efficiency (27), especially by redirecting patients away from the largest hospitals (28). Investing in healthcare infrastructure in mid-tier cities could yield higher returns and support more balanced service provision.

Fourth, the more pronounced effects appeared in higher-value procurement transactions (top 50%), likely reflecting strategic investments rather than routine purchases. This pattern suggested that civilian hospitals responded to structural shifts in demand by undertaking substantial capital upgrades. Our findings implied that systemic shocks like healthcare reform tended to trigger more intensive responses when institutions anticipated sustained, long-term changes. To support such adaptation, financing mechanisms, such as targeted grants and dedicated infrastructure funds, should be considered to facilitate large-scale strategic procurement.

Fifth, military-related specialties (e.g., trauma care) experienced earlier procurement increases, while non-military fields (e.g., pediatrics) responded more gradually. This staggered adjustment suggested an initial focus on filling core health service gaps, followed by gradual expansion into broader domains. Future reforms should account for this dynamic and incorporate phased transition planning. Displaced demand may not reallocate uniformly or immediately and may require staged support for capacity building in both core and adjacent medical domains. Complementary measures, such as training programs, inter-hospital collaboration, and expanded referral networks, can help smooth the transition for both healthcare providers and patients.

Finally, while our findings highlighted the adaptability of civilian healthcare providers, potential long-run trade-offs had to be acknowledged. Restrictions on military hospitals can hinder the development of military-specialized medical expertise, such as trauma surgery and epidemic response, which had historically advanced standards in both military and civilian healthcare (21). Although our study did not detect such long-term effects due to the limited post-reform observation window and data constraints, this remained an area of concern. Military healthcare reform should strike a balance between cost-efficiency, combat readiness, and the preservation of medical knowledge. Civilian-military integration can help maintain expertise in critical specialties, but only if accompanied by strategies to maintain clinical training environments and long-term professional development pathways.

### 4.2 Limitation and conclusion

This study has a few limitations. First, although public procurement contracts were mandated to be disclosed online, reporting-particularly in earlier years-may have been incomplete. We addressed this concern with year fixed effects, and as long as under-reporting was not systematically correlated with military hospital distribution, our findings should have remained valid. Second, as our data began in 2015, the same year the reform was launched, we lacked a pre-reform baseline. Nonetheless, the staggered implementation allowed us to capture dynamic treatment effects over time. Future research could extend our work using additional retrospective data to address these limitations.

In conclusion, China's military reform served as a natural experiment, revealing how civilian healthcare systems adapt to institutional shocks. China's healthcare system underwent many significant reforms in recent years, such as the county and township health sector integration (29) and pharmaceutical drug regulation (30). Our study explored another important reform and emphasized the need for coordinated planning between military and civilian systems to ensure that efforts to improve efficiency do not unintentionally undermine broader public health objectives.

## Data Availability

Publicly available datasets were analyzed in this study. This data can be found here: https://www.ccgp.gov.cn China's Government Procurement Database.

## Footnotes

1 Recent reforms have introduced fixed government payments for general practitioners in some regions, but higher-level hospitals largely function as described.

2
http://www.ccgp.gov.cn

3
https://fuwu.nhsa.gov.cn

4
https://www.yicai.com

5 We excluded city fixed effects because our key variable of interest, the number of military hospitals, is time-invariant and would be absorbed by city fixed effects.

6 We also tested for heterogeneity standard geographic divisions (northeast, east, central, west) and found significant effects only in the northeast. The contrast between military-based and standard geographic groupings underscored the importance of the military's command-based implementation structure, which likely shaped the heterogeneous impact of the reform.

7
https://www.nmpa.gov.cn
